# Biocompatible Hydrogel-Based Liquid Marbles with Magnetosomes

**DOI:** 10.3390/ma17010099

**Published:** 2023-12-24

**Authors:** Rafał Bielas, Tomasz Kubiak, Matus Molcan, Bernadeta Dobosz, Michal Rajnak, Arkadiusz Józefczak

**Affiliations:** 1 Faculty of Physics, Adam Mickiewicz University in Poznań, Uniwersytetu Poznańskiego 2, 61-614 Poznań, Poland; kubiakt@amu.edu.pl; 2 Institute of Experimental Physics, Slovak Academy of Sciences, Watsonova 47, 040 01 Košice, Slovakia; molcan@saske.sk (M.M.); rajnak@saske.sk (M.R.); 3 Institute of Physics, Faculty of Physics, Adam Mickiewicz University in Poznań, Uniwersytetu Poznańskiego 2, 61-614 Poznań, Poland; benia@amu.edu.pl; 4 Faculty of Electrical Engineering and Informatics, Technical University of Košice, Letná 9, 042 00 Košice, Slovakia

**Keywords:** liquid marbles, turmeric particles, magnetosomes, magnetotactic bacteria, agar, hydrogel, EPR spectroscopy

## Abstract

Liquid marbles are widely known for their potential biomedical applications, especially due to their versatility and ease of preparation. In the present work, we prepared liquid marbles with various cores composed of water, agar-based hydrogels, magnetic fluids, or non-aqueous substances. As a coating material, we used biocompatible particles of plant origin, such as turmeric grains and *Lycopodium* pollen. Additionally, we provided marbles with magnetic properties by incorporating either magnetosomes or iron oxide nanoparticles as a powder or by injecting another magnetic fluid. Structures obtained in this way were stable and susceptible to manipulation by an external magnetic field. The properties of the magnetic components of our marbles were verified using electron paramagnetic resonance (EPR) spectroscopy and vibrating sample magnetometry (VSM). Our approach to encapsulation of active substances such as antibiotics within a protective hydrogel core opens up new perspectives for the delivery of hydrophobic payloads to the inherently hydrophilic biological environment. Additionally, hydrogel marbles enriched with magnetic materials showed promise as biocompatible heating agents under alternating magnetic fields. A significant innovation of our research was also the fabrication of composite structures in which the gel-like core was surrounded without mixing by a magnetic fluid covered on the outside by the particle shell. Our liquid marbles, especially those with a hydrogel core and magnetic content, due to the ease of preparation and favorable properties, have great potential for biomedical use. The fact that we were able to simultaneously produce, functionalize (by filling with predefined cargo), and manipulate (by means of an external magnetic field) several marbles also seems to be important from an application point of view.

## 1. Introduction

Liquid marbles (LMs) are droplet systems that are of interest to material scientists owing to their potential applications and straightforward fabrication [1,2], which is essential for fundamental research. These particle-covered droplets are distinguished from Pickering droplets due to their placement in air rather than in liquid. Typically, they are formed by rolling droplets on a bed of particles, a process sometimes referred to as the ‘easiest experiment in science’ [3]. While their basic formation is quite simple, LMs still open new perspectives for interesting research, particularly when incorporating new materials as their components. Therefore, the literature reveals LMs stabilized by various solid particles, mainly with hydrophobic properties [4], including polymer, organic, and soft particles.

The broad range of explored stabilizers resulted in some unexpected applications. For example, LMs were used in experiments resembling a billiard game [5], where marbles, treated as billiard balls, were observed colliding under the influence of an external magnetic field. Rong et al. demonstrated the use of LMs with solid-liquid hybrid particles for efficient CO_2_ capture and removal [6]. Patchy and Janus LMs were also fabricated [7], combining multiple types of particles into a single system. Such heterogeneous structures not only exhibit aesthetic appeal but, above all, offer varied functions through their distinct components. The millimeter size of LMs and ease of preparation make them an excellent platform for testing complex concepts within the so-called ‘lab-on-droplet’ framework, such as mini-reactors [8,9] and other miniaturized systems. Particularly in biomedicine, LMs could be used in this context by utilizing various responsive agents, for example, magnetic nanoparticles, synthesized or obtained from organisms such as bacteria. To the best of our knowledge, there is no reported use of magnetosomes (MTs)—magnetic nanoparticles from specialized bacteria—in LMs. However, such a situation offers the potential benefits of incorporating MTs in LMs, which are isolated from external environments by a protective particle layer.

Magnetosomes (MTs) have been the subject of extensive research since the late 20th century, as modern nanotechnology increasingly values nanoparticles of biological origin equipped with natural biocompatible layers. MTs are magnetic nanoparticles inherently encapsulated by a bacterial membrane. This type of coating is particularly advantageous, as it reduces the need for additional treatment of the magnetic particles, e.g., covering them with surfactants in order to minimize agglomeration and modify their interaction with living tissues and organisms [10]. Thanks to the developed methods for cultivating bacteria and extracting nanoparticles from them, MTs were rigorously tested for various applications. These include serving as carriers for anticancer drugs [11,12] and contrast agents in magnetic resonance imaging (MRI) [13,14]. In the context of the reported drug delivery systems, the use of larger carriers to transport MTs may expand their biomedical applications. Challenges such as the limited half-life and stability of MTs, as well as the aggregation prior to delivery at the target site, can be addressed through this approach [15]. Encapsulating MTs within protective carriers can mitigate these risks, allowing for controlled release at the desired location. As we will show in the present work, the adaptable and protective nature of LMs as containers could open up new possibilities when combined with the unique features of MTs.

From a fundamental perspective, LMs exhibit behaviors distinct from both bulk liquids and uncoated droplets, offering advantages in terms of reducing the risks of contamination and material loss during transportation [16,17]. For instance, unlike microfluidic systems, LMs offer the flexibility to move liquids in multiple directions [17]. This versatility becomes evident when LMs are exposed to external stimuli. Regardless of them, the particle layer of LMs protects the inner liquid, enabling, e.g., mixing the liquid in the core. This can significantly enhance internal processes, as demonstrated by experiments with droplets covered by fumed silica particles [18]. In the mentioned case, the iodine–starch colorimetric reaction was easily evaluated by direct observation. The apparent advantage of performing such tests inside LMs is the possibility of using transparent particles for optical control of the internal processes. However, much more crucial is the small volume of LM that facilitates the processes, such as the growth of microalgae, due to a large surface-to-volume ratio [9]. In fact, LMs were shown to be more effective for cultivating algae than traditional bottles and flasks. Additionally, LMs have a higher effective viscosity compared to pure droplets, influencing their spread on surfaces [19]. This property is particularly useful when LMs are intentionally broken to release active substances from their core. Additionally, the deformation rate of LMs was shown to be higher since the presence of stabilizing particles reduces the surface tension with increasing volume of the LM [20]. Simultaneously, the presence of a particle layer at the droplet surface brings important consequences—the evaporation process is still significant because of the pores between particles (mostly micrometers in size in most of the reports) [21]. Although some permeability might be considered as an unwanted side effect, it can also be advantageous in certain applications. For instance, evaporation induced by high temperatures in LMs with polyethylene terephthalate (PET) and NaCl crystals led to the formation of hollow shells [22]. Capsules fabricated in this way from LMs as templates could carry pharmaceutics, enzymes and probiotics similarly to polymer capsules that became more and more common in targeted delivery of active agents [23].

Contrary to what their name might suggest, LMs can carry more than just liquid cores. Hydrogels, which closely resemble soft tissues, also adhere to solid surfaces, which makes them difficult to manipulate unless they are entrapped in a particle layer [24]. This led to the development of liquid marbles with gel-like cores, sometimes referred to as ‘hydrogel marbles’ [25], opening up new possibilities in biomaterial development. For example, Oliveira et al. explored hydrophobic hydrogel LMs for in vitro tissue creation based on their previous work with floating LMs [16,26]. The inherent similarity of hydrogels to human tissues in terms of their properties, such as density, led other groups to test hydrogels and LMs. Instead of using the hydrogel as the core of LM, Vadivelu et al. used LM as carriers to form templates for growing 3D tissue models, such as cell toroids, within armored droplets [27]. Similarly, LMs based on gelatin and coated with polytetrafluoroethylene (PTFE, also known as Teflon) powder were used to preserve cells at low temperatures [28]. On the other hand, the gel structure of LMs also offers an advantage in terms of stability, protecting them from rapid disintegration due to evaporation, a common challenge to LMs. Another significant development involves the transport of hydrophobic substances. Typically, these poorly soluble substances are intended for delivery to the inherently hydrophilic environment of tissues. LMs that encapsulate hydrophobic substances within a hydrogel core, and are further surrounded by a biocompatible layer, represent an innovative approach for cargo transportation. This could be potentially important for applications involving biological media as the hydrogel core effectively contains the hydrophobic substances, while the biocompatible outer layer ensures compatibility and minimal adverse interactions with biological environments. This is a notable advancement over traditional water-based LMs, which struggle to incorporate hydrophobic agents. Furthermore, the natural elasticity of hydrogels not only enhances the stability of LMs but also influences their fabrication methods.

In the present study, we have successfully prepared LMs containing MTs and other magnetic materials, using *Lycopodium* and turmeric powder as stabilizing agents. We explored various ways to manipulate these LMs: transporting them using static, external magnetic fields, treating them with elevated temperatures under alternating magnetic fields, and re-functionalizing them by adding magnetic materials to their cores. We thoroughly tested and discussed these diverse treatment scenarios to demonstrate the wide range of manipulation techniques available for LMs formed on the basics of hydrogel (agar) and water droplets enriched with magnetic material.

## 2. Materials and Methods

### 2.1. Particles and Liquids

Bio-based particles used as stabilizers for LMs were commercially available as medical or dietary products. We sourced fine turmeric powder from Dary Natury (Grodzisk, Poland) and hydrophobic *Lycopodium* powder (derived from the *Lycopodium clavatum* plant) from Nanga Co. in Złotów, Poland. To explore the magnetic functionalization of LMs, we used iron (II, III) oxide nanoparticles (IONPs) with the size of 20–30 nm and provided by IoLiTec Ionic Liquids Technologies GmbH (Heilbronn, Germany).

For the core of the LMs, we chose, among others, distilled water or agar-based hydrogels. The plate count agar powder (product no. 88588) was obtained from Sigma-Aldrich Co. (St. Louis, MO, USA). To demonstrate the versatility of LMs, we filled some of them with other liquids. Commercial linseed oil was purchased from Oleofarm Co. (Wrocław, Poland). Clarithromycin, dispersed in the linseed oil, served as the model antibiotic drug. Moreover, we used HEPES (4-(2-hydroxyethyl)-1-piperazineethanesulfonic acid) and PEG400 (Sigma-Aldrich Co. St. Louis, MO, USA) as alternative liquid phases in certain experiments.

In addition to the previously mentioned materials, our research also employed two types of water-based magnetic fluids. The first contained spherical magnetite-based clusters (sMAG), with an average particle size of approximately 200 nm, prepared according to the method outlined in [29]. The concentration of magnetic material was 1.62 mg/mL. The second contained iron oxide particles (~14 nm in size) coated with perchloric acid (MNPs@PA) [30]. This fluid had a magnetic particle concentration of 75 mg/mL.

### 2.2. Preparation of Magnetosomes

As a source of MTs, we used nanoparticles isolated from the magnetotactic bacteria *Magnetospirillum magneticum* sp. *AMB-1*, grown in controlled laboratory conditions. This type of bacterium, within its cell, synthesizes MTs, which consist of magnetite nanoparticles (Fe_3_O_4_) bound together by an organic envelope and forming a chain-like structure. A detailed procedure for isolating magnetosomes can be found in [31]. In brief, the isolation involved multiple steps: sonication to disrupt the bacterial cells, centrifugation for separating the components and magnetic decantation for extracting the magnetosomes. During these steps, we consistently purified the sample using HEPES buffers. The HEPES buffer was set to a pH of 7.4, close to physiological pH, making it suitable for various biological experiments. The initial outcome was a HEPES-based suspension of magnetosomes. To further purify the MTs, we repeated these steps, gradually replacing the HEPES buffer with distilled water, resulting in a final suspension of magnetosomes in water. The concentration of the MT suspension in the HEPES buffer was 3 mg/mL, while in distilled water, it was 0.8 mg/mL.

### 2.3. Preparation of Magnetosome-Based Liquid Marbles with Aqueous and Hydrogel Cores

To prepare the hydrogel core of the LMs, we first measured the required quantities of distilled water and agar powder. The agar was then dissolved in boiling water. This approach slightly differs from our previous works, where we used agar gel as tissue-mimicking phantoms, e.g., [32,33]. Here, we utilized the agar–water solution in its liquid state, at about 30 °C, before it gelled. The agar concentration in the gel was maintained at 5% *w/w*.

The formation procedure of the LMs was consistent regardless of the type of core used. This process followed the method presented in [29] and was similar to standard protocols for fabricating LMs. In short, a certain amount of the liquid phase—whether water, agar-in-water solution, or HEPES—was deposited drop-wise using a micropipette on a bed of substrate particles spread on a wax-coated base. The droplets were then mechanically rolled to coat them evenly with particle powder. Additionally, in order to enhance the functionality of the LMs for potential biomedical applications, we employed a custom setup for filling marbles with the predefined payload. The device was based on a movable automatic multi-channel pipette mounted on a special metal holder, as shown in Figure S1 in Supplementary Materials. The position of the pipette could be adjusted mechanically, which guaranteed precision (elimination of handshaking) and allowed for controlled filling of up to 8 marbles at the same time.

### 2.4. Characterization of Particles and Liquid Marbles

To analyze the size of the particle stabilizers and the formation process of the LMs, we utilized an optical microscope equipped with a digital camera (AM7115MZTL, Dino-Lite Europe Co., Almere, The Netherlands). The captured pictures and movies were processed and analyzed on a PC. In this way, we successfully examined various coatings and controlled experiments that involved manipulating LMs. These manipulations included puncturing, filling with different liquids, and functionalizing the shell. Additionally, we employed ImageJ software, ver. 1.8.0 (National Institutes of Health, Bethesda, MD, USA) to assist in manually calculating the particle size distribution from microscopy images. Furthermore, magnetosome nanoparticles were characterized using Transmission Electron Microscopy (TEM) imaging. This was performed with a JEM-1400 microscope (JEOL Ltd., Tokyo, Japan) at an accelerating voltage of 120 kV.

The magnetic properties of the MTs were determined using a vibrating sample magnetometer (VSM). We placed 30 µL of MTs-in-water dispersion in a sealed tube and loaded it into the magnetometer, which was coupled with a cryogen-free superconducting magnet from Cryogenic Ltd. (London, UK) at a temperature of 295 K. The magnetic moment was measured in the low-field range from −0.1 T to 0.1 T, and specific magnetization was calculated as the ratio of the magnetic moment to the mass of the sample.

Additionally, the MTs dispersed in various liquids, along with iron oxide nanoparticle (IONPs) magnetic powder and spherical magnetite-based cluster (sMAG) water-based magnetic fluid, were examined using an X-band electron paramagnetic resonance (EPR) spectrometer, EPR/ENDOR EMX-10, (Bruker Co., Billerica, MA, USA). The settings for these EPR experiments included a modulation amplitude of 0.5 mT, a time constant of 40.9 ms, and a sweep range of 650 mT centered at 333.8 mT. All measurements were conducted at room temperature.

In several experiments, we utilized an alternating magnetic field (AMF) to induce a temperature increase in the magnetic LMs. For this purpose, we employed the EASYHEAT induction heating system (Ambrell Co., Rochester, NY, USA). This system converted a standard line frequency (50 Hz) to a high-frequency signal (around 355 kHz), generating an AMF intensity of 16.2 kA/m.

## 3. Results and Discussion

### 3.1. Properties of Magnetosomes and Stabilizing Particles

**Figure 1:**
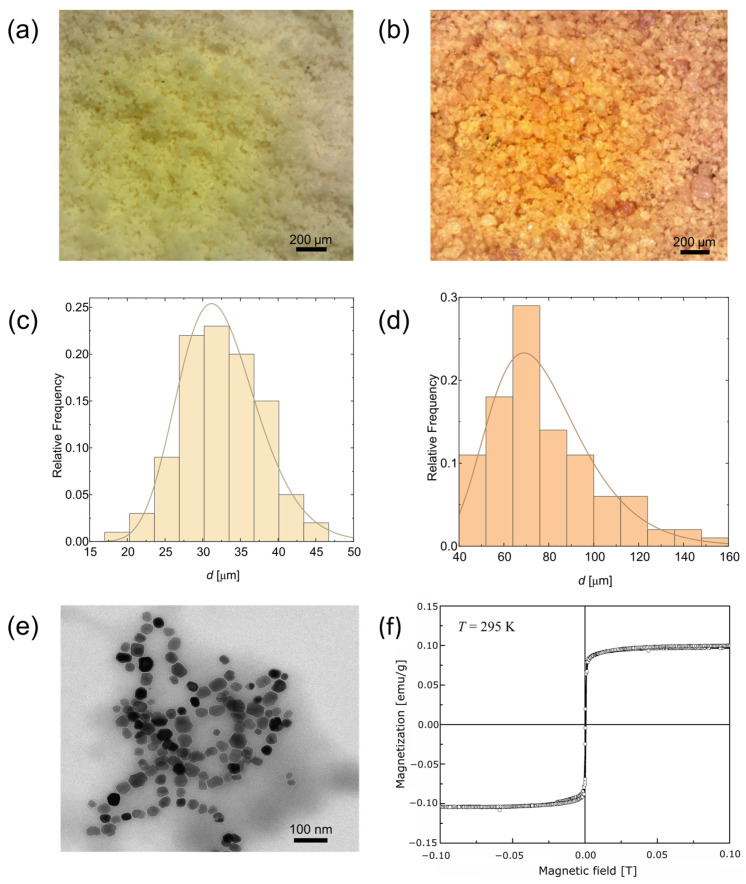
Characterization of materials used in the experiments. Optical microscopy images of (**a**) *Lycopodium* and (**b**) turmeric particles. Particle size distributions obtained from optical microscopy imaging for (**c**) *Lycopodium* and (**d**) turmeric particles with fitted log-normal functions. (**e**) Transmission electron microscopy (TEM) images and (**f**) magnetization curve of a magnetosome suspension in water measured at 295 K for the concentration of 0.8 mg/mL.

The response of LMs to external stimuli, such as static and time-varying magnetic fields, is significantly influenced by the properties of the materials comprising the LMs. We conducted a thorough characterization of both the stabilizing particles and the MTs encapsulated within the particle coating of the droplets. The physical attributes and size of the turmeric and *Lycopodium* particles were examined using a digital optical microscope, and the magnetic responsiveness of the MTs was assessed through VSM measurements, as depicted in Figure 1.

Our analysis revealed that both turmeric and *lycopodium* particles were in powdered form, without any tendency to form aggregates, making them suitable for creating a particle bed necessary for the fabrication of LMs. The average size of *Lycopodium* particles was 30 µm. While other reports show that *Lycopodium* pollen particles are not spherical but rather irregular in shape, this was not clearly observed in optical microscopy images shown in Figure 1a. Turmeric powder particles were clearly less spherical and more varied in size, with a mean dimension of about 70 µm. Due to their bio-origin and proposed use in both oral and topical medical applications, both *Lycopodium* [34] and turmeric demonstrated biocompatibility. The presence of curcumin, a major component of turmeric powder, further enhances the utility of this material, given its antibacterial, antifungal, and anticancer properties [35], although the limited bioavailability of curcumin is also a concern [36].

MT nanoparticles naturally form chain-like structures (as illustrated in Figure 1e), a characteristic that stems from the physiology of the bacteria used as their templates. Although the natural membrane enveloping these nanoparticles is not distinctly observable as shell lines around irregularly shaped particles in the size of tens of nanometers, it is indicated by the thin gaps between these particles. The membrane plays a crucial role in influencing the organization of nanoparticles within the depicted structure. The magnetization curve obtained from the water suspension of MTs (Figure 1f) exhibits no significant hysteresis loop. This observation suggests that the particles, encapsulated by a biocompatible layer, exhibit superparamagnetic behavior. This property, typical for Fe_3_O_4_, indicates that these MTs should demonstrate magneto-responsiveness.

From a practical point of view, determining the actual composition of magnetic particles within a sample is crucial. EPR spectroscopy can be an effective technique for identifying the specific type of iron oxide present in a sample. EPR spectroscopy is a recognized method for examining samples containing unpaired electrons. The splitting of energy levels called the Zeeman effect, occurs for specimens placed in an external magnetic field. When the energy of microwave radiation corresponds to the energy difference between the splitted energy levels Δ*E*, the resonance condition is fulfilled, and the EPR spectrum is observed that can be expressed by the following relation:

Δ*E* = *hv* = *gμ*_B_*B*, (1)

where *g* is the spectroscopic splitting factor characterizing a given paramagnetic center, *μ*_B_ is the Bohr magneton, *B* is the induction of the external magnetic field, and *ν* is the microwave frequency. The experimentally determined *g*-factor value for a specific paramagnetic center differs from the *g* value for a free electron due to the contribution of the orbital angular momentum and different local fields of atoms or molecules.

**Figure 2:**
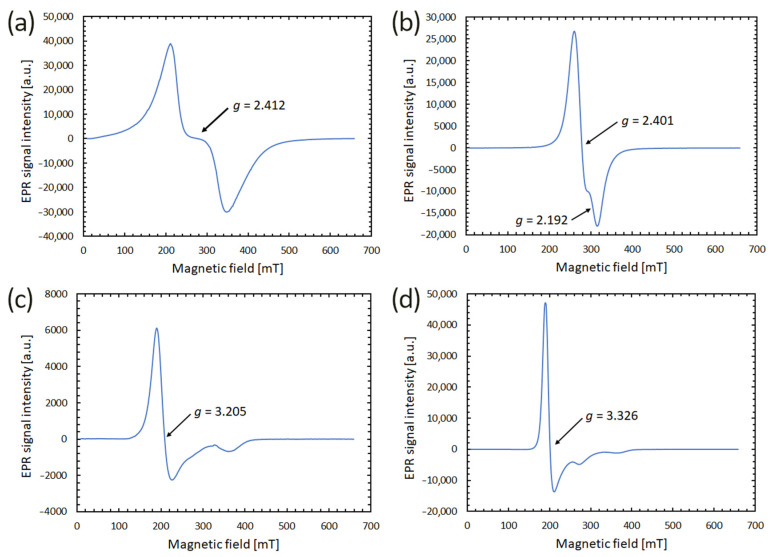
EPR spectra of magnetic nanoparticles: (**a**) IONP powder; (**b**) magnetic fluid containing spherical magnetite-based clusters sMAG, (**c**) MTs in water; (**d**) MTs in HEPES. Values of *g*-factors for the main lines are marked in the individual panels.

Figure 2 illustrates the EPR signal intensity as a function of magnetic field strength for four distinct magnetic samples. These specimens include IONP powder, sMAG dispersion in water, and MTs dispersed in both water and HEPES. Their characteristics derived from these EPR spectra are detailed in Table 1.

The spectra of all four samples were dominated by a distinct signal coming from the magnetic core of the nanoparticles. The intensity of this line varied depending on the specimen, indicating differences in the concentration of magnetic material. IONP powder exhibited the strongest magnetic properties, which was confirmed not only by EPR measurements but also by the high response to magnetic manipulation of marbles containing “magnetic caps” made of this material (as we will discuss in Section 3.5). On the other hand, the much weaker signal from MTs dispersed in water indicated a lower content of magnetic material, which corresponded to the observed poor response of marbles with such cores in the external magnetic field. The significantly lower signal amplitude for magnetosomes in water compared to magnetosomes in HEPES may be related to the method of extraction of these particles from bacteria. Samples were purified using HEPES buffer, which was later gradually replaced with distilled water, which diluted the magnetic material.

Other parameters of EPR spectra, such as *g*-value and peak-to-peak EPR linewidth Δ*B_pp_* also differed within the examined materials, as shown in Table 1. The broad spectrum of IONP powder came from microscopic clusters chaotically distributed in the sample. The shape of the resulting spectrum was the envelope of the EPR lines originating from individual, differently oriented centers.

In the case of the EPR spectrum of magnetic fluid containing spherical magnetite-based clusters (sMAG) apart from the main line (*g* = 2.401), we can see weaker components (*g* = 2.192) with the resonance field shifted towards the greater values. The sMAG spectrum may indicate the presence of two types of centers with different Fe(II)/Fe(III) ratios in the crystal structure. Therefore, we probably observe the trace of maghemitization (oxidation) of the magnetite nanoparticles in the sample. For MT specimens, we recorded a strongly asymmetric EPR signal. This indicates anisotropy due to a wide distribution of spin packets in the applied magnetic field. Compared to other studies on MTs performed using the EPR technique [37,38], we observed higher *g* values of the main line of our samples. A significant increase in the effective *g*-factor, above the value close to 2.0, observed for stoichiometric magnetite, indicated the oxidation of magnetite grains in MTs used in the experiment [38].

Interestingly, differences between the spectra of MTs dispersed in water and HEPES were observed. Based on the knowledge that each MT contains magnetite crystals enclosed within a lipid bilayer membrane, the dissimilarity in the spectra indicated possible variations in the interactions between magnetic centers and their surroundings. EPR studies of laboratory-synthesized Fe_3_O_4_ nanoparticles dispersed in different media proved that even small changes in the environment can affect the magnetic properties of these nanoparticles [39]. The parameters of the EPR spectra were, therefore, different for functionalized magnetite nanoparticles dispersed in water, organic solvents [40], whole human blood [41], standardized (sodium alginate and calcium chloride) hydrogel [42], and agar phantoms [33]. One of the reasons for these variations may be the different share of the oxidized Fe^3+^ compared to Fe^2+^ atoms on the outer layer of the particles. According to the literature, the strong radial anisotropy experienced by surface spins is responsible for the shift and broadening of the spectra [43]. The cause is the influence of the electric field gradient resulting from the lack of outer oxygen in the first coordination octahedron surrounding the magnetic ions.

One can notice that the procedure of producing and storing affects the final properties of MTs, which was also confirmed by the recorded EPR signals. The differences visible in the spectra of magnetic liquids (containing both MTs and magnetic clusters, sMAG) at different stages of their life span may be the subject of further research in the future.

It is also worth stressing that despite its insightful capabilities, EPR spectroscopy is not a widely employed technique for characterizing magnetic samples. The assessment of the properties is particularly significant for particles like MTs, which are not synthesized in the same way as single magnetic nanoparticles (e.g., IONPs) or magnetic clusters (sMAG) but are instead extracted from biological organisms. In our research, we prioritized testing these particles using EPR spectroscopy before incorporating them into the core of LMs, a process that will be detailed in the next sections.

### 3.2. Behavior of Turmeric-Based Liquid Marbles

**Figure 3:**
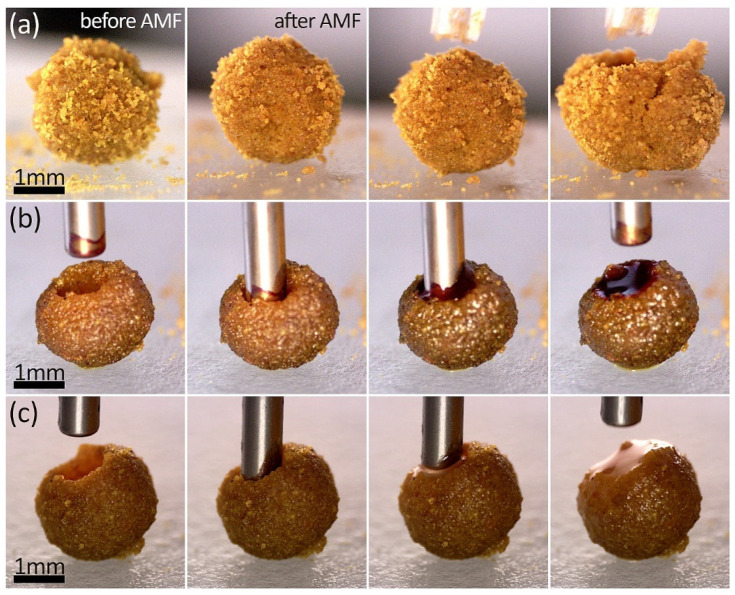
Optical microscopy images of the droplet-based structures with the shells made of turmeric powder. (**a**) LM was created based on a droplet of MTs in water and then exposed to AMF for 5 min. Crushing the turmeric shell using a pipette tip indicated absorption of the magnetic liquid by the coating and partial evaporation of water. (**b**) Refilling of the structure resulted from MTs in water-based turmeric LM with MNPs@PA magnetic fluid. (**c**) Refilling of the structure resulted from MTs in water-based turmeric LM with clarithromycin dispersion in linseed oil.

Our investigation of LMs began with an evaluation of turmeric powder as a stabilizing coating. This type of particle has already been utilized for the creation of colloidal capsules [44,45], where it proved to be effective as a stabilizer for Pickering droplet-based capsule formation. Figure 3a shows a single LM with a water core coated with turmeric particles, both before and after exposure to an AMF. The polydispersity of the particles led to a somewhat uneven layer, contrasting with results we had obtained previously using homogeneously sized polystyrene particles [29]. Despite the application of an AMF that increased the temperature, the rough surface of the LM remained largely unchanged. However, an interesting observation was made when the LM was punctured with a pipette tip. Then, the entire structure failed to sustain the water droplet, which was coated with turmeric particles.

In our previous work, we found that the exposure of LMs with a magnetic core to a high-frequency AMF accelerates the evaporation process, leading to the decomposition of the LM [29]. Here, the turmeric particle shell can efficiently absorb the aqueous core, effectively leaving behind a magnetic shell containing the incorporated MTs. While the application of AMF improved the absorption of the water content, it did not significantly change the external appearance of the LMs.

Following the simple formula presented in [46], one can determine the percentage sphericity, *s*, of the LM:

(2) $$s=\frac{shorter\;length\;of\;LM}{longer\;length\;of\;LM}\times 100.$$

The *s* value for the LMs calculated based on Equation (2) depicted in Figure 3 varies from 88.9% for LM before refilling with MNPs@PA magnetic fluid to 98.7% for turmeric LM after AMF application. It demonstrates that, despite the apparent lack of smoothness in the particle layer, turmeric particles effectively coated the droplet surface. This resulted in the formation of relatively spherical LMs. The ability to maintain a spherical shape is particularly vital in the context of scaling up the production process and ensuring consistent quality control of the fabricated structures. In this context, the size distribution of stabilizing particles also plays a role, as narrow size distribution tends to result in the formation of a single layer of individual particles around the liquid cores, provided that the average size of these particles is not excessively large. For example, hydrophobic polystyrene particles, when coated and larger than 20 μm, were observed to form a more regular hexagonal monolayer compared to smaller particles [4]. This difference in particle size significantly influences the mechanical resistance of the structure during transport.

Structures formed by turmeric particles after absorbing water can then be efficiently filled with specific liquids, as demonstrated with MNPs@PA magnetic fluid (shown in Figure 3b) and dispersion of the antibiotic clarithromycin in biocompatible linseed oil (illustrated in Figure 3c). The observed behavior of the particle layer after gaining the new liquid core indicated that the characteristic elasticity of the structure was not lost in opposition to the sudden loss of elasticity when the force by the pipette tip was applied, as shown in Figure 3a. In both instances, we created LMs containing MTs, which migrated from the core to the coating following the evaporation of the liquid from the base droplet. This transition provides the LMs with new functionalities. From the point of view of further transport of MTs, it is noteworthy that they do not necessarily need to be encapsulated within the liquid core. Instead, they can be integrated with turmeric particles that have been moistened by the adsorbed droplet. Consequently, such prepared LMs could serve as versatile carriers for various substances, with the potential for activation and manipulation thanks to the unique properties of the incorporated MTs.

### 3.3. Behavior of Liquid Marbles Stabilized with Lycopodium Pollen

**Figure 4:**
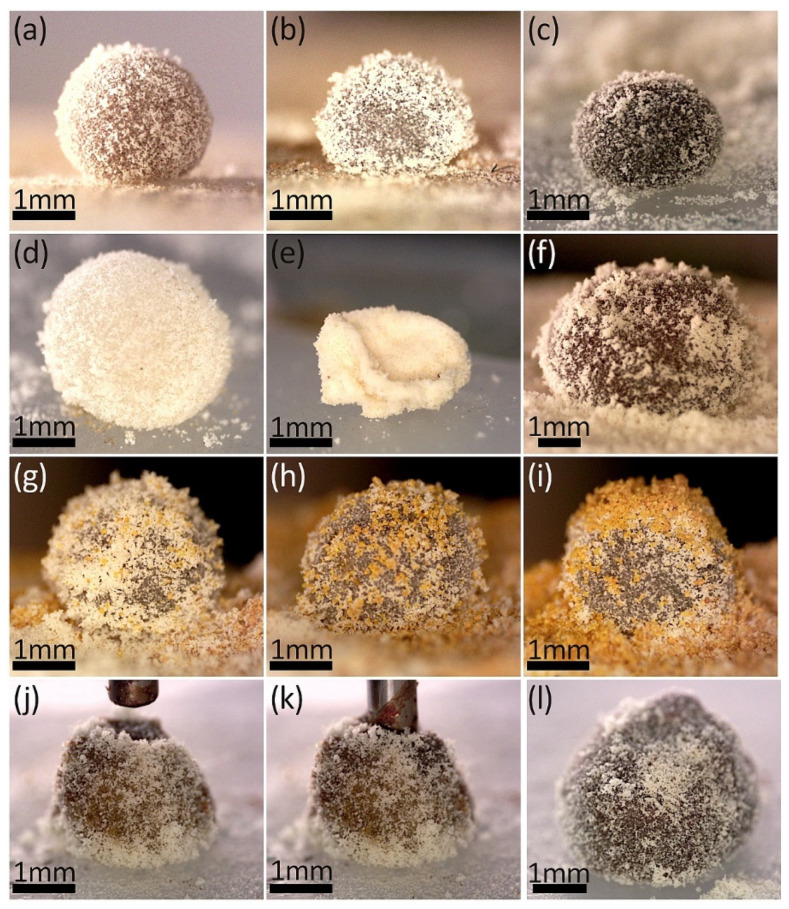
LMs with the shells made of *Lycopodium* pollen: (**a**) with MTs-in-water core; (**b**) with MTs-in-HEPES core; (**c**) with MNPs@PA magnetic fluid core; (**d**) with water-based agar gel core; (**e**) with water-based agar gel core after resting for two days; (**f**) with MNPs@PA magnetic fluid-based agar gel core; (**g**–**i**) with MTs in HEPES core, and shell sprinkled with turmeric powder to various extent. (**j**–**l**) The procedure for filling the double-layer (turmeric powder and *Lycopodium* pollen) shell with MNPs@PA magnetic fluid.

Similar to the turmeric particles, biocompatible and bio-originated *Lycopodium* pollen was employed as a particle coating for LMs. Optical images in Figure 4a–c display LMs containing suspensions of MTs in water and HEPES, as well as the MNPs@PA magnetic fluid. In each case, we successfully produced stable LMs with complete particle coverage. Due to the smaller size and more uniform distribution of the *Lycopodium* particles compared to turmeric powder, the surface of these LMs is smoother. However, the sphericity of the LMs shown in Figure 4a–c falls within the range of 73.7–89.0%, which indicates that LMs formed with *Lycopodium* pollen have a tendency to be more quasi-spherical in shape. Similar to the observations made with turmeric particles, the distance between the liquid core and the surface on which the LM is placed is affected by the size of the stabilizing particles. With larger particles, the capillary forces are stronger, which contributes to enhanced stability. Interestingly, a similar effect of improved stability was also observed, with smaller particles forming multi-layers at the interface, which helps maintain the structural integrity of the LM [4]. When polydisperse particles were used to form LMs, mechanical robustness was higher than for particles with a narrow size distribution [17]. Another aspect is the tendency for deflation due to the evaporation of the liquid core, which also depends on the size of particles and their structure [47] and could contribute to the differences in the sphericity values for *Lycopodium*-based LMs. When compared to the turmeric-based LMs, it is not without significance that the turmeric particles absorbed the aqueous content.

As mentioned in the Introduction, LMs with gel-like cores open up new perspectives for developing materials, especially for the controlled delivery of drugs and active substances. To explore this potential, we prepared agar-based LMs stabilized with *Lycopodium* particles. Figure 4d presents an optical image of an LM containing a hydrogel structure as its core. Despite the presence of stabilizing particles and the internal gel-like structure, such an LM collapsed after two days (as shown in Figure 4e), resulting in a hard, non-elastic residual structure. In contrast, Figure 4f presents a magnetic hydrogel LM. Due to the inclusion of magnetic material, this type of LM could potentially be moved via magnetophoresis without direct contact, as demonstrated previously [5,48,49].

While *Lycopodium* particles alone yielded stable LMs, as observed with turmeric powder, we also explored the feasibility of creating LMs using both particle types (as depicted in Figure 4g–i). Initially, a droplet containing a dispersion of MTs in HEPES was entrapped within a *Lycopodium* particle coating. Subsequently, varying amounts of turmeric powder were sprinkled on top to form an irregular dual-particle coating. This simple method resulted in LMs with MTs irregularly covered by two different types of particles. An alternative approach involved creating LMs with a double layer of coating (shown in Figure 4j–l), which could then be filled with magnetic fluid. Literature suggests that such a dual-layer coating can enhance the mechanical properties of LMs, particularly due to the contrast in particle sizes, making the LMs more resilient to deformation [50]. While larger particles can form regular hexagonal-like patterns, smaller ones could fill pores between them and reinforce the stiffness of the whole.

The shape of LMs seems to be significantly influenced by the degree of gelation of their core during the rolling process on the particle bed. This gelation level affects the sphericity of LMs in a manner somewhat similar to the ‘arrested coalescence’ observed in Pickering emulsions. In Pickering emulsions, the particles at the interface sometimes become jammed due to excessive coverage. A similar phenomenon has been observed in LM when subjected to electric fields [51]. Regarding the LMs presented in Figure 4, their sphericity values range from 0.65 to 0.92, as detailed in Table S1 of the Supplementary Materials. This variation in sphericity can be attributed to the excess particles adsorbed on the surface of the gel-like core, which results from the irregular shape the core gains during the rolling process. The distinct behavior of the gel-like core during rolling, influenced by the degree of gelation, has not been extensively explored in existing literature.

### 3.4. Multi-Step Functionalization of Hydrogel-Based Liquid Marbles with Magnetosomes

As shown above, we were able to fabricate LMs with cores composed of either water or water-dissolved agar powder. If applicable, our marbles can possess magnetic properties arising from the presence of magnetosomes (MTs) within the aqueous core or from MTs introduced into the LM’s interior via injection. The capability to easily functionalize LMs (even multiple times) significantly enhances their utility in a broad range of biomedical applications.

**Figure 5:**
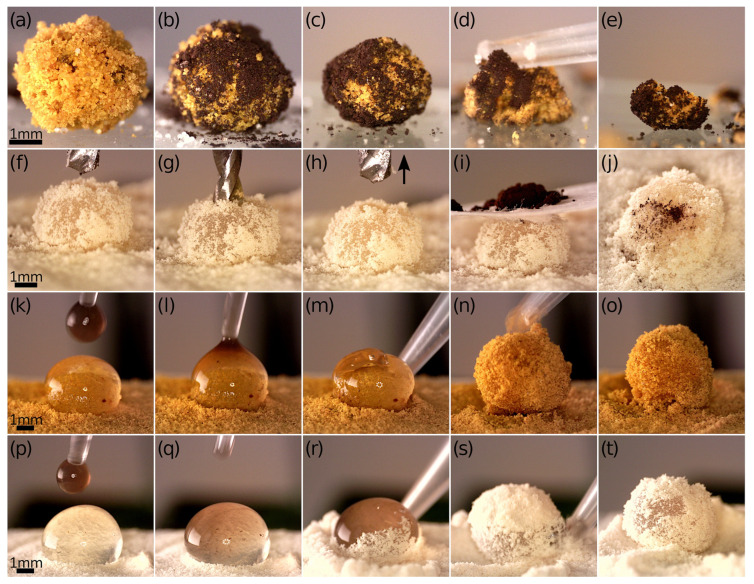
Optical microscopy images of LMs and different strategies for adding magnetic materials. (1) **Sprinkling with magnetic particles**: (**a**) LM with MTs in water-based agar gel core and turmeric powder shell; (**b**) LM after sprinkling with IONP powder; (**c**) sprinkled LM after 5 min in AMF; (**d**) testing the strength of the structure by crushing it with a pipette tip; (**e**) mechanically crushed fragments of the shell indicated that the particles were stuck together. (2) **Drilling and filling with IONP powder**: (**f**) LM with water-based agar gel core and shell made of *Lycopodium* pollen; (**g**) puncturing the LM using a small drill; (**h**) pulling out the drill (indicated by black arrow) revealed a hole in the center of the LM; (**i**) magnetite powder was poured through the hole into the center of the marble; (**j**) top view of the marble: magnetic material inside the drilled hole. (3) **Pouring with MTs dispersion and covering with turmeric particles**: (**k**) water-based agar gel core deposited on turmeric bed; (**l**) pouring MTs-in-water droplet onto the gel core; (**m**–**o**) coating with turmeric powder by rolling. (4) **Pouring with MTs dispersion and covering with *Lycopodium* pollen**: (**p**) water-based agar gel core deposited on *Lycopodium* bed; (**q**) pouring MTs-in-water droplet onto the gel core; (**r**–**t**) coating with *Lycopodium* pollen by rolling.

In Figure 5, we showed LMs containing agar and MTs in their core, both before and after the addition of extra magnetic nanoparticles, which were either in the form of powder (IONPs) or additional suspension of MTs in water. This process led to the creation of patchy agar-based LMs (Figure 5a–c) with a dual magnetic nature both inside and outside. The presence of two different types of magnetic materials, distinguishable by EPR measurements (as seen in Figure 2), results in two key consequences. Firstly, adding extra magnetic nanoparticles increases the heat generated when the LMs are exposed to an AMF, as illustrated by the LMs functionalized with IONP powder in Figure 5c. This is similar to the previously discussed impact of AMF on the water-absorbing capability of turmeric granules (Figure 3) and also affects the behavior of agar-based LMs. Secondly, despite MTs being considered as a source of heat, the temperature increases recorded in our experiments were not significant. This observation aligns with the results from studies of magnetic hyperthermia in agar tissue-mimicking phantoms, where the temperature elevation from MTs was also not very significant, mostly due to the low actual concentration of magnetic material extracted from magnetotactic bacteria [52,53]. The biocompatible nature of MTs, combined with the better magnetic heating performance of IONP powder, could result in a change in properties.

The presence of an uncoated agar core still allowed for the incorporation of additional magnetic materials by pouring MTs-in-water droplets onto it. As depicted in Figure 5k–o, a portion of MTs suspended in water was successfully added to the hydrogel droplet. Following the coalescence of these droplets, the resulting double-magnetic structure was coated with turmeric powder to form the LM. This technique was efficient regardless of the type of stabilizing particles used; *Lycopodium* pollen also served effectively as a particle bed (Figure 5p–t). In the future, such strategies could be employed to precisely control the dosage of magnetic biocompatible materials, like MTs, and concurrently, the active substances associated with them within the carriers like LMs.

### 3.5. Controlled Transport of Hydrogel-Based Liquid Marbles with Magnetosomes

The ability to scale up the production and utilization of LMs is essential for developing their practical applications. For instance, Castro et al. proposed a simple sliding ramp to increase the production rate up to tens of LMs/minute. So far, it has been based on a manual creation using a mechanical pipette, allowing the formation of Janus structures by coalescing droplets [54]. In turn, Krov et al. applied the setup with a moving particle bed using a small belt conveyor and obtained a fabrication rate of up to 50 LMs/s [55]. Another strategy is presented in the up-to-date work of Tenjimbayashi, who designed the special hydrophobic mesh covered by particles that allowed the production of multiple LMs at the same time by immersing the mesh in a water pool [56].

**Figure 6:**
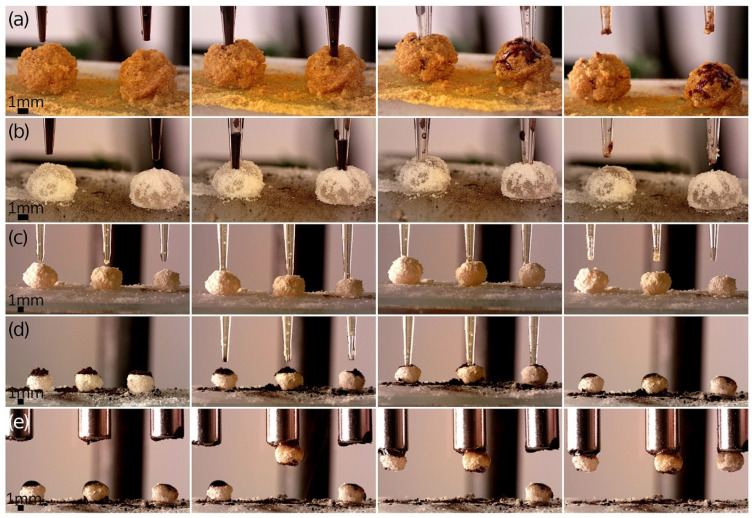
Optical microscopy images showing simultaneous functionalization of multiple LMs. (**a**) Filling turmeric-coated agar-based LMs with MNPs@PA magnetic fluid. (**b**) Filling *Lycopodium*-coated agar-based LMs with MNPs@PA magnetic fluid. (**c**) Filling *Lycopodium*-coated agar-based marbles with clarithromycin dispersion in linseed oil. (**d**) Enrichment of *Lycopodium*-coated agar-based LMs with magnetic caps IONP powder and filling them with clarithromycin dispersion in linseed oil. (**e**) Transport of magnetite-capped *Lycopodium*-coated agar-based LMs using neodymium magnets.

Our methods for a facile functionalization of hydrogel LMs by the addition of extra magnetic material (such as IONP powder or MTs) were also used for multiple LMs. Simultaneous filling of multiple LMs (lined up in a row) with the desired specimen was possible thanks to the use of a multi-channel device (see Figure S1 in Supplementary Materials). Each panel in Figure 6 demonstrates multiple LMs that were functionalized either by injecting magnetic fluid (MNPs@PA) into the core or by adding IONP powder on the top to form ‘magnetic caps.’ These caps not only covered the injection site but also prevented the content from spilling out of the LM.

Perhaps the most intriguing finding, shown in Figure 6e, is the potential of the ‘magnetic caps’ to act as additional attractors for the external static magnetic field. We previously discussed the limited capacity of MTs (constituting also the core in LMs in Figure 6) to generate a significant magnetic heating effect. Their low concentration, a result of the formation process and safety considerations for biotechnological applications, also limited the magneto-responsiveness of the LMs. Although the MTs in the LM’s core could interact with the external magnetic field, this interaction primarily caused the bulk motion of the entire LM and often led to its disintegration upon direct contact with magnetic surfaces rather than controlled transport. Therefore, the addition of ‘magnetic caps’ could be a highly beneficial and straightforward enhancement. Moreover, the amount of magnetic material within these ‘magnetic caps’ controlled in a facile way how strongly the LMs were drawn to applied neodymium magnets. This explains the order in which the LMs in Figure 6e were attached to the magnets (one after another).

### 3.6. Magnetically-Assisted Manipulation of Liquid Marbles for the Fabrication of Composite Structures

**Figure 7:**
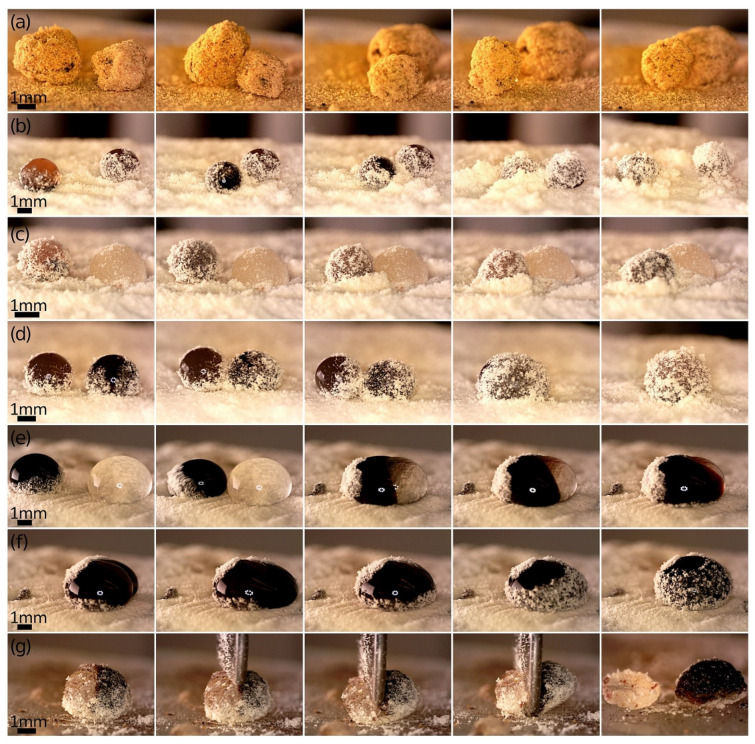
Optical microscopy images showing the magnetic manipulation of LMs. (**a**) Magnetically driven movement and mutual collisions (‘magnetic billiards’) of LMs with MNPs@PA fluid-based agar gel core and turmeric powder shell after 5 min in AMF. (**b**) External magnetic field-induced motion of the sMAG droplet (originally on the left) resulted in its coverage with *Lycopodium* pollen and collision with the MTs-in-water droplet without coalescence. (**c**) Magnetically controlled displacement of the LM with sMAG core and *Lycopodium* shell and its interaction with the LM made of PEG400 covered with *Lycopodium* pollen. (**d**) Magneto-coalescence of two droplets: MTs-in-water (left) and sMAG-in-water (right) partially covered with *Lycopodium* pollen. (**e**,**f**) Under the influence of an external magnetic field, MNPs@PA magnetic fluid surrounded the stationary water-based agar ellipsoid, created an outer layer on it. (**g**) The *Lycopodium* powder-coated two-phase structure consisting of agar and MNPs@PA magnetic fluid was subjected to AMF (5 + 15 min) and then mechanically bisected along the phase boundary.

Beyond the controlled transfer of LMs from one location to another via an external magnetic field, the displacement and magneto-coalescence of LMs were also possible. This type of manipulation is similar to ‘magnetic billiards’, a concept previously reported in the literature. It involves moving and merging LMs using magnetic forces and offers a novel approach to manipulating these structures in various applications [5,57]. However, as demonstrated in our research, similar magnetic interactions can also occur with non-aqueous and gel-like LMs. When these LMs are completely coated with multi-layers of biocompatible particles, as shown in Figure 7a, they undergo mutual collisions without coalescing. In the mentioned experiments, magnets were positioned beneath the particle bed. This demonstration indirectly confirmed that the LMs were effectively entrapped in a particle shell, as the magnetic forces caused them to move and collide without merging.

In another experiment, we observed the behavior of uncovered magnetic droplets containing different magnetic materials (MTs vs. sMAG) when subjected to a static magnetic field. These droplets, shown in Figure 7b, bounced upon contact but did not coalesce. The sMAG droplet driven by the magnetic field became partially covered while rolling. Its collision with the MTs-in-water droplet resulted in the momentum transfer. Consequently, the second drop began to roll. One should remember that the direct contact of uncovered liquids can mostly initiate the coalescence, although there are reports on the coalescence of LMs upon sufficient momentum during collisions [58] or sufficient voltage used to charge LMs [59]. Instead, the droplets presented in Figure 7b exhibited competitive behavior, ultimately leading to both structures being entirely covered by *Lycopodium* pollen. In another scenario presented in Figure 7c, LM with sMAG core and *Lycopodium* shell interacted with LM made of PEG400 and also covered with *Lycopodium* pollen. When these marbles came into direct contact, they did not merge. Rather, they were involved in a kind of ‘wrestling’ (trying to push each other), or they ‘buried’ themselves in a bed of particles. This tendency of LMs to either coalesce or merely remain in contact without merging appears to be primarily influenced by the degree of coverage of both droplets with particles at the moment of contact or the presence of large pores in the coating, as we showed for Pickering emulsion droplets coalesced under electric fields [60,61]. However, in the case of LMs, a more detailed examination of the merging process uncovered additional factors influencing their behavior. While it is known that the likelihood of coalescence in colliding LMs depends on the balance between kinetic energy and surface energy, the size of the particles constituting the LM surface also plays a significant role. Specifically, it was observed that smaller particles tend to facilitate coalescence [58]. The coalescence process of LMs resembles the merging of pure droplets, but due to the presence of the protective particle layer surrounding the LMs, the difficulty of achieving coalescence is greater [62]. In Figure 7d, the particle coverage was less complete, and the droplets under the influence of a static magnetic field coalesced, which was a unique example of the magnetically driven formation of a double-magnetic LM (MTs combined with sMAG). Importantly, the sphericity calculated for LM prepared via rolling the coalesced droplet on the particle bed (Equation (2)) was rather high (88.1%), demonstrating that even facile magnetic manipulation could lead to stable double-magnetic LMs.

Using neodymium magnets, we were also able to form an outer layer of MNPs@PA magnetic fluid around the gel-like core. This fluid layer moved from one side of the LM to the other, as demonstrated in Figure 7e and Video S1 in Supplementary Materials, seemingly without mixing the two phases. As a result, we obtained a structure similar to the ‘composite LMs’ described by Roy et al., who reported forming aqueous LMs coated with silica particles and filled with silicone oil in the interstitial spaces [63]. In our experiment, the two-phase structure was further gradually enveloped by *Lycopodium* pollen as a result of the movement of the liquid around the hydrogel core driven by the magnetic field (Figure 7f). Additionally, we investigated the effect of temperature increase caused by magnetic heating under an AMF within the magnetic core of these composite LMs. Cutting the LM along the phase boundary revealed that there was no mixing between the two phases, highlighting the distinctiveness of this composite structure, as shown in Figure 7g.

Even though our methods for refilling hollow LMs have been demonstrated specifically for MTs, they are not restricted to them alone. It is possible that other responsive agents, such as those with conjugated functionalized molecules for active targeting, could also be effectively incorporated within LMs serving as cargo carriers. The presence of solid particles on the surface of LMs can be advantageous in slowing down evaporation, which could be particularly beneficial in drug delivery applications, such as skin treatments improved with heating. This system would allow for controlled release rates of active substances. Additionally, the size of the particles is a critical factor, as smaller particles have a larger surface-to-volume ratio.

As previously mentioned, MTs show promise as biocompatible heating agents under AMF, and the use of LMs can enhance their utility due to the ease of transport offered by these structures. However, LMs might also function as phase change materials, capable of storing thermal energy in a reversible manner [64] instead of leading to the evaporation of the liquid core and making hollow structures.

## 4. Conclusions

In this work, the LMs stabilized with bio-based turmeric and *Lycopodium* particles and containing MTs incorporated into their core exhibited numerous interesting features important from the application point of view. In particular, the agar-based LMs demonstrated unique behavior when filled with additional magnetic fluids and during magneto-coalescence with aqueous magnetic LMs. Hydrogel LMs, mostly spherical in shape and functionalized with magnetic materials, exhibited enhanced responsiveness to external magnetic fields, facilitating their transportation. The inherent elasticity of the hydrogel cores contributed to the overall stability of the LMs while still enabling extensive functionalization. It is worth emphasizing that we were able to transport multiple LMs simultaneously, taking a significant step toward the scalability of the usage of LMs.

We also studied the formation of composite LMs, comprising a hydrogel core surrounded by a liquid phase, further stabilized by a layer of solid particles. Such magnetic LMs, with both gel and liquid phases, along with other functionalized variants of LMs, present promising platforms for delivering active agents. Utilizing MTs, which are enveloped in a natural biocompatible membrane, LMs can target specific sites, potentially through magnetic heating or other external stimuli, accompanied by magnetic field-driven transportation. Furthermore, our approach offers a novel solution for delivering hydrophobic active substances, often limited by solubility issues, by encapsulating them within a protective hydrogel core. This makes them suitable for cargo delivery to inherently hydrophilic biological environments like tissues. The variety of manipulation and functionalization techniques presented in our work paves the way for developing platforms that leverage MTs and their derivatives for targeted delivery. The combination of LMs with MTs opens up new possibilities in the design of materials, particularly in enhancing drug delivery systems such as skin lotions, where targeting can be facilitated through a magnetic field.

## Figures and Tables

**Table 1:** Parameters of the main line of EPR spectra of magnetic sample.

Sample	*g*-Value	$\Delta B_{pp}$ [mT]	Asymmetry Parameter
IONP powder	2.412 ± 0.013	136.6 ± 1.1	1.295 ± 0.001
sMAG dispersed in water	2.401 ± 0.007	55.3 ± 1.1	1.490 ± 0.001
MTs dispersed in water	3.205 ± 0.012	36.9 ±1.1	2.694 ± 0.001
MTs dispersed in HEPES	3.326 ± 0.013	21.6 ± 1.1	3.444 ± 0.002

## Data Availability

The data will be available on request.

## Supplementary Materials

The following supporting information can be downloaded at: https://www.mdpi.com/article/10.3390/ma17010099/s1, Video S1: The process of formation of composite magnetic fluid−hydrogel marble. The additional description consists of Figure S1: The scheme of setup for filling/refilling of LMs with multi-channel pipette and Table S1: The summary of sphericity values for LMs fabricated in the experiments as well as Figure S2: Sphericity values for all LMs presented in this work.
