# Supplementary material for: Biocompatible Hydrogel-Based Liquid Marbles with Magnetosomes
Source: Materials (Basel). 2023 Dec 24;17(1):99. doi: 10.3390/ma17010099 (PMC10779466; doi:10.3390/ma17010099)
Supplement: Supplementary file 1 [file materials-17-00099-s001.zip › materials-2779066-supplementary.pdf]

materials

Supporting information for

Biocompatible Hydrogel-Based Liquid Marbles with Magnetosomes

A Custom Setup for Filling Liquid Marbles with the Predefined Payload

The custom device was based on an automatic pipette mounted on a special metal holder as schematically shown in Figure S1. The position of the pipette can be adjusted mechanically, which guarantees precision and allowed for controlled filling up to 8 marbles at the same time. Such a setup was used to show the simultaneous functionalization of multiple liquid marbles (LMs) by filling them with magnetic fluid (Figure 6).

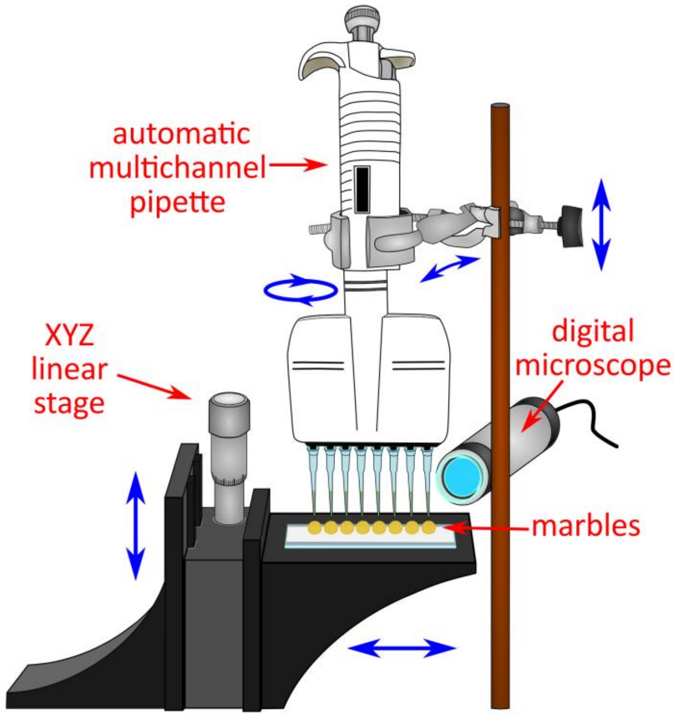

Figure S1. A scheme of a custom setup for simultaneous filling of multiple LMs (lined up in a row) with desired specimen controlled by digital optical microscope.

Sphericity Values for Liquid Marbles Fabricated in the Experiments

The liquid marbles (LMs) presented in this work were characterized by their sphericity (s) defined as shown in Equation 2. The higher % value, the more spherical shape of the imaged LMs. The summary of all LMs shown in the paper is presented in Table S1 and Figure S2 that indicates how s values vary.

Table S1. Sphericity (*s*) values for LMs presented in the paper calculated based on Equation 2. The terms: left, right, center refer to the position of the LM shown in the corresponding figure.

No. of LM	Description of LM	Sphericity [%]
1	Figure 3a	0.97887
2	Figure 3a	0.98658
3	Figure 3b	0.88889
4	Figure 3c	0.9635
5	Figure 4a	0.89041
6	Figure 4b	0.75172
7	Figure 4c	0.73723
8	Figure 4d	0.92
9	Figure 4f	0.65174
10	Figure 4g	0.94156
11	Figure 4h	0.86624
12	Figure 4i	0.91837
13	Figure 4j	0.89706
14	Figure 4l	0.72989
15	Figure 5a	0.8
16	Figure 5b	0.85714
17	Figure 5c	0.8125
18	Figure 5f	0.68056
19	Figure 5o	0.92308
20	Figure 5u	0.73529
21	Figure 6a (left)	0.83516
22	Figure 6a (right)	0.84884
23	Figure 6b (left)	0.81944
24	Figure 6b (right)	0.75
25	Figure 6c (left)	0.67273
26	Figure 6c (center)	0.72917
27	Figure 6c (right)	0.80488
28	Figure 7a (left)	0.84524
29	Figure 7b (right)	0.8806
30	Figure 7d (right)	0.88095
31	Figure 7f (right)	0.71

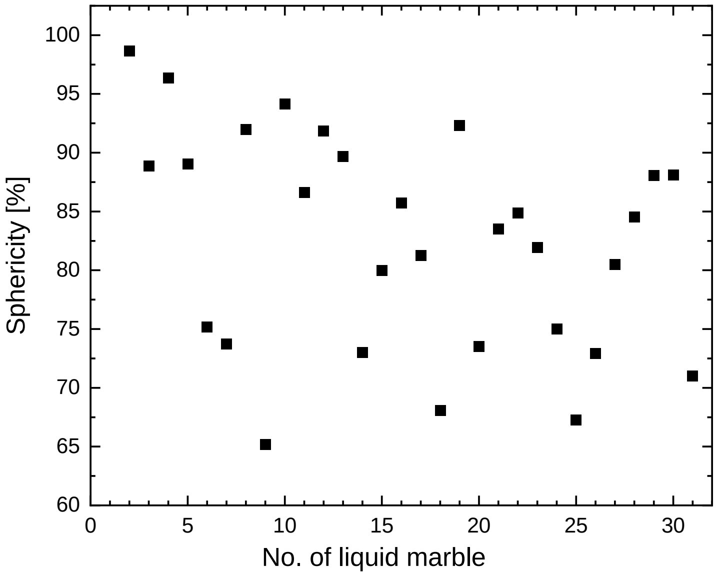

Figure S2. Sphericity values (Equation 2) for all LMs presented in this work. Number of liquid marble is aligned with the Table S1.
